# Ouabain Affects the Expression of Activation Markers, Cytokine Production, and Endocytosis of Human Monocytes

**DOI:** 10.1155/2014/760368

**Published:** 2014-05-08

**Authors:** Mariana Pires Teixeira, Vivian Mary Rumjanek

**Affiliations:** Laboratório de Imunologia Tumoral, Instituto de Bioquímica Médica Leopoldo de Meis, Universidade Federal do Rio de Janeiro, Centro de Ciências da Saúde, H_2_003 Cidade Universitária, Ilha do Fundão, 21941-590 Rio de Janeiro, Brazil

## Abstract

Ouabain is a steroid capable of binding to and inhibiting Na^+^,-K^+^-ATPase. Studies have demonstrated some actions of ouabain on immune cells, which indicated both pro- and anti-inflammatory properties of this molecule. Nevertheless, its effects on human monocytes are still poorly understood. Thus, the present work investigated effects of ouabain in the activation and function of human adherent monocytes. Our results show that there is an increase in intracellular calcium levels already 5 minutes following monocyte treatment with 10^−7^ M of ouabain. Furthermore, monocytes expressed increased amounts of surface activation markers such as CD69, HLA-DR, CD86, and CD80 and also presented an augmented endocytic activity of dextran-FITC particles after 24 hours of culture in the presence of ouabain. However, monocytes treated with ouabain did not have an increased stimulatory capacity in allogeneic mixed leukocyte reaction. Ouabain-treated monocytes produced higher levels of IL-1**β** and TNF-**α** as reported before. A novel observation was the fact that ouabain induced IL-10 and VEGF as well. Collectively, these results suggest that ouabain impacts monocyte activation and modulates monocyte functions, implying that this steroid could act as an immunomodulator of these cells.

## 1. Introduction

Ouabain was initially described as a compound extracted from plants, such as* Acocanthera ouabaio* and* Strophanthus gratus*, and has been used for the treatment of congestive heart failure due to its positive ionotropic effect on cardiac muscle [1]. However, in 1991, an endogenous analogue, biologically, structurally, and immunologically indistinguishable from the ouabain extracted from plants, was discovered in human plasma [2].

Nowadays, this endogenous ouabain is considered part of a new class of steroid hormones [1]. Under physiological conditions, levels of endogenous ouabain are relatively low and range between 10^−9^ and 10^−11^ M, whereas pharmacological levels are around 10^−7^ M. In stress situations, like physical exercise, these endogenous levels can also reach 10^−7^ M in humans [3–5]. Additionally, increased levels of ouabain have also been associated with different pathological situation, such as hypertension, cardiac dysfunctions, chronic renal failure, and preeclampsia [1, 4, 6–10]. Ouabain is secreted by hypothalamus and adrenal glands and is capable of binding to Na^+^,K^+^-ATPase, inhibiting Na^+^ and K^+^ transport and triggering the activation of several cell signaling pathways [11–13].

Though the physiologic and pathophysiologic roles of ouabain are not well established, ouabain was shown to affect the immune system [14, 15] and to have anti-inflammatory properties [16, 17]. Notwithstanding, ouabain was shown to modulate the release of proinflammatory cytokines by mononuclear cells. In fact, it was demonstrated that treatment of these cells with 10^−7^ M of ouabain promotes an increase of IL-1*β*, TNF-*α* secretion [18–20]. Furthermore, our group has demonstrated that after incubation with ouabain, a significant percentage of monocytes expressed lower levels of mCD14 and this appears to involve transactivation of EGFR and activation of p38MAPK [21]. Nevertheless, the effect of ouabain on other cell surface molecules or even the impact of ouabain on monocyte function has not been addressed yet.

Monocytes are crucial for immune responses, since they can perform effector functions such as antigen presentation, endocytosis, and the production of a diverse array of cytokines [22]. Thus, the aim of the present work was to verify whether ouabain was able to alter the activation state and function of monocytes, in an attempt to understand a possible role of this molecule on human monocytes.

## 2. Methods

### 2.1. Cell Separation

Human peripheral blood samples were obtained from healthy volunteers (<40 years old) and separated by Ficoll-Histopaque (GE, Sweden) density gradient centrifugation (400 ×g, 30 minutes). After separation, mononuclear cells were collected, washed three times by centrifugation at 200 ×g for 10 minutes with phosphate buffered saline (PBS), and resuspended in culture medium RPMI 1640 (Sigma, USA) supplemented with 10% fetal calf serum (FCS) (Gibco, USA), inactivated at 56°C for 1 hour. The study was approved by the Ethics Committee of the Hospital Universitário Clementino Fraga Filho-UFRJ, and all volunteers gave written informed consent to this work.

### 2.2. Cell Culture

Mononuclear cells were plated in 24-well plates (TPP, Switzerland) at a concentration of 2 × 10^6^ cells/well in a final volume of 1 mL and incubated for 2 hours in an atmosphere of 5% CO_2_ at 37°C. After this period, cells were washed twice with PBS for the removal of the excess of lymphocytes that, unlike monocytes, remain in suspension. Finally, culture medium RPMI 1640 supplemented with 10% FCS was added to the culture.

Following the 2 hours of adhesion, cells were further incubated in the presence or absence of ouabain (OUA) (Sigma, UK) in an atmosphere of 5% CO_2_ at 37°C.

### 2.3. Monocyte Staining and Flow Cytometric Analysis

To study surface molecules expression, cells were washed with PBS, removed with a cell scraper, and labeled with human FITC-conjugated anti-CD14 (BioLegend, USA) or PE-conjugated anti-CD14 (BD Pharmingen, USA) and PE-conjugated anti-HLA-DR (BD Pharmingen, USA), PE-conjugated anti-CD86 (BD Pharmingen, USA) or FITC-conjugated anti-CD80 (BD Pharmingen, USA), and FITC-conjugated anti-CD40 (BD Pharmingen, USA) or PE-conjugated anti-CD69 (BD Pharmingen, USA) for 30 minutes at 4°C.

Cells were then washed twice with PBS + FCS 5% (200 ×g, 7 minutes) and resuspended in the same solution in the cold. Fluorescence measurements were performed using a FACScan or a FACSCalibur flow cytometer (Beckton, Dickinson and Company, USA). Fluorescence signals for no less than 10,000 cells were recorded in the monocyte gate, which was determined according to cell size and complexity parameters. Using these parameters, approximately 98% of the cells in this gate were CD14^+^. Data analyses were performed using Summit v4.3 software (Dako, USA).

### 2.4. Intracellular Calcium Measurement

After the 2-hour adhesion and removal of nonadherent cells, the remaining cells were incubated with 5 *μ*M of Fluo-3AM (Sigma, USA) for approximately 10 minutes at 37°C. This was followed by the addition of 10^−7^ M of ouabain to the culture and monocytes were further incubated for 5, 15, and 30 minutes. Adherent cells without ouabain were used as control. For a positive control, thapsigargin was added to the culture (data not shown). Finally, cells were washed with ice-cold PBS and fluorescence intensity was analyzed by flow cytometry. Fluorescence signals for no less than 10,000 cells were recorded in the monocyte gate.

### 2.5. Allostimulatory Capacity

After 24 hours of incubation in the absence or presence of 10^−7^ M of ouabain, monocytes were collected and cocultured with lymphocytes from a second donor for 5 days in 96-well round-bottom plates (final volume of 200 *μ*L). The number of lymphocytes in this mixed culture was fixed at 10^5^ cells per well in all conditions evaluated. For a 1 : 1 (monocyte : lymphocytes) ratio, 10^5^ monocytes were added to the culture and, for a 1 : 10 (monocyte : lymphocytes) ratio, 10^4^ monocytes were added to the culture. We also cultured monocytes (10^5^, as used in 1 : 1 ratio mixed cultures and 10^4^ cells/well, as used in 1 : 10) and lymphocytes (10^5^ cells/well, as used in both ratio mixed cultures) alone as control proliferation. For a positive control, lymphocytes were cultured with 5 *μ*g/mL of PHA (data not shown). Cell proliferation was assessed after these 5 days by [^3^H]thymidine incorporation. For that, an additional 18-hour pulse with 0.5 *μ*Ci of [^3^H]thymidine per well was performed. After pulse, cells were collected on filter papers and nonincorporated [^3^H]thymidine was removed by washing the filter papers with water. Then, these filter papers were added to scintillator vials containing scintillator fluid. [^3^H]Thymidine incorporation was measured using a liquid scintillator counter.

### 2.6. Cytokine Detection

After 24 hours of incubation in the absence or presence of 10^−7^ M of ouabain, culture supernatants were harvested and stored at −20°C until cytokine assay. Cytokine detection was performed by enzyme-linked immunosorbent assay (ELISA). Production of IL-1*β* and TNF-*α* was measured using Human IL-1*β* Matched Antibody Pairs for ELISA and Human TNF-*α* Matched Antibody Pairs for ELISA (Bender MedSystems, Austria). Production of IL-10 and TGF-*β*1 was measured using Human IL-10 ELISA and Human TGF-*β*1 ELISA (Bioscience, USA). Production of VEGF was measured using Human VEGF Elisa Development kit (PeproTech, USA). Assays were performed according to each manufacturer instruction.

### 2.7. Endocytic Activity

After 24 hours of incubation in the absence or presence of 10^−7^ M of ouabain, cells were incubated with 1 mg/mL FITC-conjugated dextran (Sigma, USA) for 1 hour at 37°C or 4°C (for control endocytic activity). Cells were then washed with ice-cold PBS to remove free dextran particles and fluorescence was analyzed by flow cytometry. Fluorescence signals for no less than 10,000 cells were recorded in the monocyte gate. Data analyses were performed using Summit v4.3 software (Dako, USA).

### 2.8. Statistical Analysis

The statistical analysis of the data was determined by two-tailed paired *t*-test or repeated measures ANOVA with Dunnett's posttest, with the exception of cytokine production that was determined by Wilcoxon matched pairs test. Values where *P* < 0.05 were considered statistically significant. Most experiments were performed at least 3 times with distinct subjects and, when this was not done, the number of experiments is indicated in the Figure legend.

## 3. Results

### 3.1. Ouabain Affects the Expression of Surface Molecules Related to Cell Activation

To investigate the impact of ouabain on monocyte activation induced by adhesion, we analyzed the expression of CD69, HLA-DR, CD80, CD86, and CD40. These molecules may be used as activation markers, since their expression may be induced or simply upregulated upon the activation of monocytes by different stimuli [23].

Our results show that HLA-DR and CD86 were already expressed by the majority of peripheral blood monocytes, whereas CD80 and CD40 were present only in a very small percentage (approximately 1%) of these cells. Also, approximately half of these peripheral blood monocytes expressed CD69.

In addition to that, we observed that monocytes cultured for 24 hours, in the absence of ouabain, expressed higher levels of HLA-DR and CD86 per cell (approximately a twofold increase) compared to monocytes in the PBMC fraction. Moreover, there was an increase in the percentage of monocytes expressing CD80 and CD40 (sixfold for CD80 and sixteenfold for CD40) following the incubation of cells for 24 hours in the absence of ouabain. On the other hand, the percentage of monocytes expressing CD69 diminished (approximately a threefold decrease) (data not shown).

**Figure 1:**
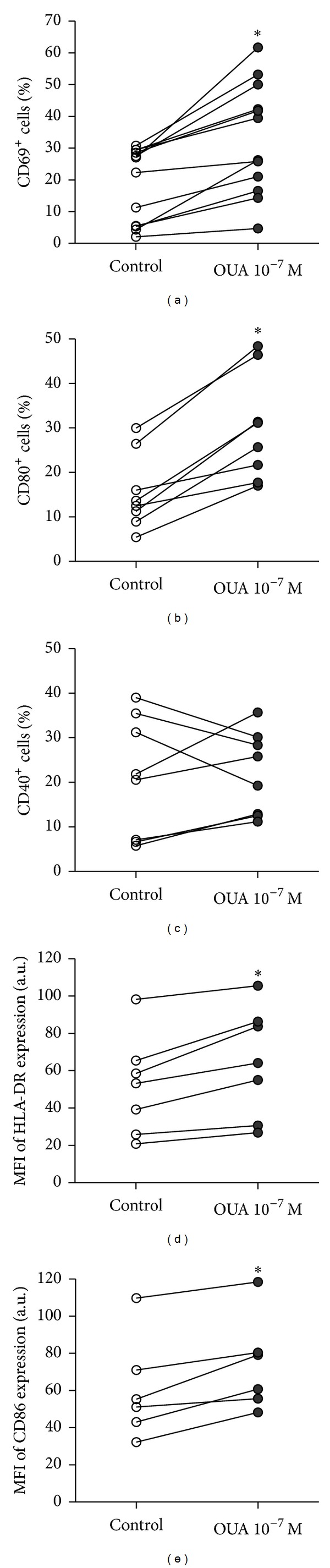
Ouabain affects the expression of CD69, HLA-DR, and costimulatory molecules on monocytes. Cells were cultured for 24 hours in the absence or presence of ouabain 10^−7^ M; thereafter, cells were incubated for 30 minutes with specific antibodies, as described in Methods. Scatter plots are shown for the percentage of CD69^+^ (a), CD80^+^ (b), and CD40^+^ (c) cells and the mean fluorescence intensities of HLA-DR (d) and CD86 (e) after 24 hours of incubation in the presence and absence of 10^−7^ M of ouabain. Each line represents the data from one specific donor. *P < 0.05 using two-tailed paired *t*-test.

Ouabain, at the concentration of 10^−7^ M, promoted an increase on the percentage of CD69^+^ monocytes (Figure 1(a)) and a very small increment on the mean density of CD69 molecules per cell (data not shown). An increase was also observed on the percentage of monocytes expressing CD80 after 24 hours of incubation with ouabain (Figure 1(b)), whereas results with CD40 were inconclusive (Figure 1(c)). Hence, ouabain-treated monocytes expressed even higher levels of HLA-DR and CD86 than control monocytes (Figures 1(d) and 1(e)).

#### 3.1.1. Ouabain Dose Response Curve

**Figure 2:**
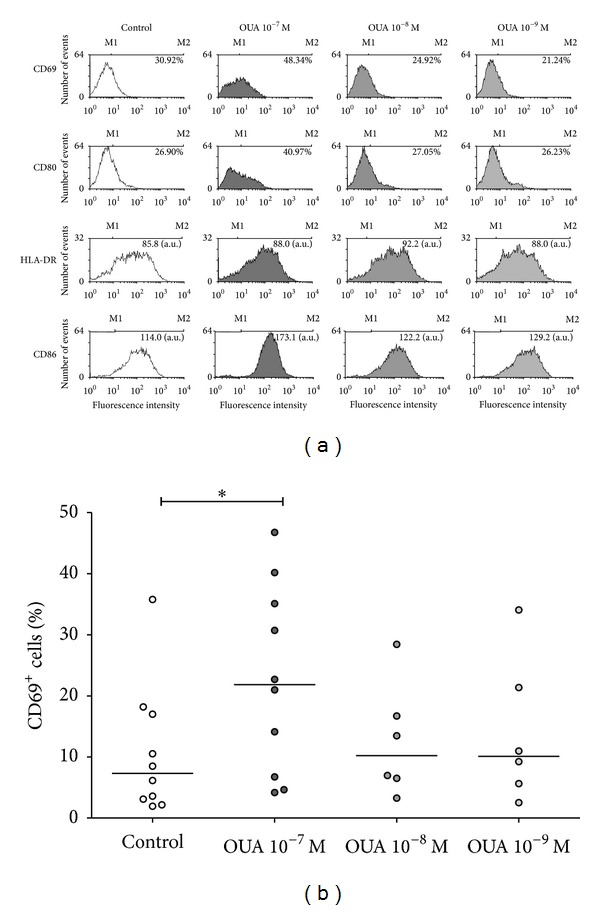
Ouabain dose response curve on the expression of activation and costimulatory molecules by monocytes. Cells were cultured in the absence or presence of 10^−7^ M, 10^−8^ M, and 10^−9^ M of ouabain for 24 hours. After 24 hours, these cells were incubated for 30 minutes with specific antibodies, as previously described. (a) Histograms show the patterns of CD69, CD80, HLA-DR, and CD86 expression on monocytes untreated or treated with different concentrations of ouabain. Values of CD69^+^ and CD80^+^ cells and mean fluorescence intensity of HLA-DR^+^ and CD86^+^ cells were also added to the histograms. Control and OUA 10^−7^ M, *n* ≥ 6; OUA 10^−8^ and 10^−9^ M, *n* = 1, with the exception of CD69 experiments (*n* ≥ 6). (b) Data are expressed as the percentage of CD69^+^ monocytes and lines refer to the median of ten (Control and OUA 10^−7^ M) or six (OUA 10^−8^ and 10^−9^ M) independent experiments. *P < 0.05 using both repeated measures ANOVA with Dunnett's posttest (when analyzing only corresponding data) and two-tailed paired *t*-test (when analyzing all data).

Ouabain concentration of 10^−7^ M is the pharmacological dosage and may also be attained after exercise. Lower concentrations, such as 10^−8^ and 10^−9^ M, have been described as capable of inducing physiological changes in other cell types and were also tested in the present work. As observed in Figure 2, the most marked differences on cell were obtained with 10^−7^ M, and this concentration was used throughout.

#### 3.1.2. Ouabain Time Course Response

**Figure 3:**
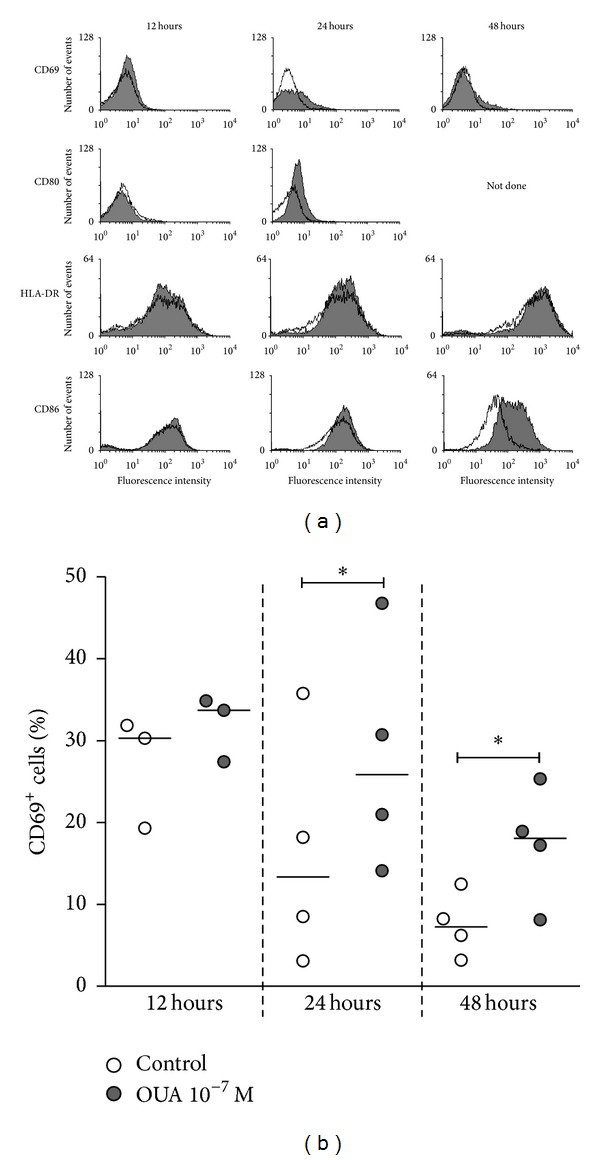
Time course of ouabain effect over the expression of activation and costimulatory molecules by monocytes. Cells were labeled with specific antibodies for 30 minutes (see Section 2) after 12, 24, and 48 hours of culture in the absence or presence of 10^−7^ M of ouabain. (a) Histograms showing the patterns of CD69, CD80, HLA-DR, and CD86 expression on monocytes untreated (empty histograms) or treated with 10^−7^ M of ouabain (gray histograms) for 12, 24, and 48 hours. 24 hours, *n* ≥ 6; 12 and 48 hours, *n* = 1, with the exception of CD69 experiments (*n* ≥ 3). (b) Data are expressed as the percentage of CD69^+^ monocytes and lines refer to the median of three (12-hour experiments) or four (24- and 48-hour experiments) independent measurements from distinct individuals. *P < 0.05 using two-tailed paired *t*-test.

Modifications induced by ouabain on cell surface molecules expressed by monocytes were studied at different time-points. A significant increase could be observed after 24 or 48 h (Figure 3).

### 3.2. Ouabain Promotes Intracellular Calcium Elevation

**Figure 4:**
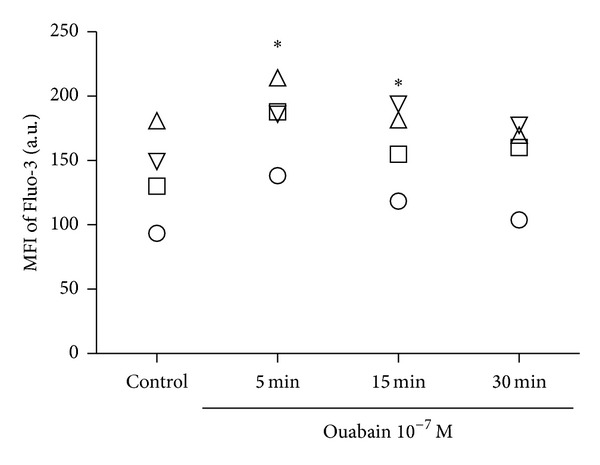
Intracellular calcium in monocytes following ouabain exposure. Cells were loaded with Fluo-3AM (5 *μ*M) for 10 minutes and then incubated with 10^−7^ M of ouabain for 5, 15, and 30 minutes. Scatter plots indicate the means of fluorescence intensity for Fluo-3 in monocytes in each condition. Equal symbols represent the data from the same individual (*n* = 4). *P < 0.05 using repeated measures ANOVA with Dunnett's posttest.

Our results suggest that 10^−7^ M of ouabain was able to modulate monocyte surface molecules expression. This specific concentration could induce an inhibition of Na^+^,K^+^-ATPase activity, which could, in turn, impact Na^+^-Ca^2+^exchanger and lead to an increase of calcium in the cytosol. A transitory increase in intracellular calcium levels was already apparent 5 minutes after 10^−7^ M ouabain exposure. Return to control levels were observed after 30 minutes (Figure 4).

### 3.3. Ouabain Does Not Influence Monocyte Allostimulatory Capacity

HLA-DR, CD86, CD80, and CD40 molecules are essential for antigen presentation. HLA-DR and these costimulatory molecules interact with their respective receptors on lymphocytes and provide the necessary signals to induce proper T cell stimulation [24]. Since ouabain was able to increase the expression of some of these molecules, we sought to evaluate the effect of ouabain on the allostimulatory capacity of monocytes.

**Figure 5:**
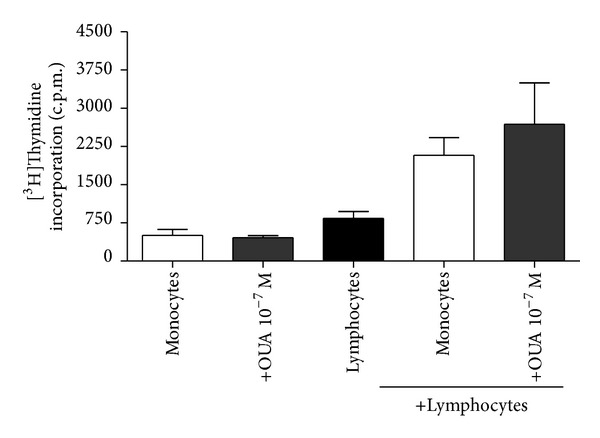
Allogeneic Mixed Leukocyte Reaction (MLR) ability of ouabain-treated monocytes. Ouabain-treated and control monocytes were cocultured with lymphocytes from a second donor using the ratio of 1 : 1 (monocyte : lymphocyte) for 5 days followed by an 18 h pulse with [^3^H]thymidine to assess the proliferative response. Results are expressed as mean c.p.m. ± SEM of four independent experiments. Each condition in these experiments was performed in duplicate.

Our results show no significant difference between the proliferation of lymphocytes cocultured with control monocytes and ouabain-treated monocytes in the proportion of 1 monocyte to 1 lymphocyte (Figure 5) or 1 monocyte to 10 lymphocytes (data not shown).

### 3.4. Ouabain Modulates Cytokine Secretion

Previous studies have shown that ouabain modulates the production of proinflammatory cytokines, such as IL-6, IL-1*β*, and TNF-*α* [18–20], by mononuclear cells. However, the role of ouabain on the production of anti-inflammatory cytokines by these cells was not addressed so far. Therefore, we next aimed to study ouabain effects on the secretion of different types of cytokines.

**Figure 6:**
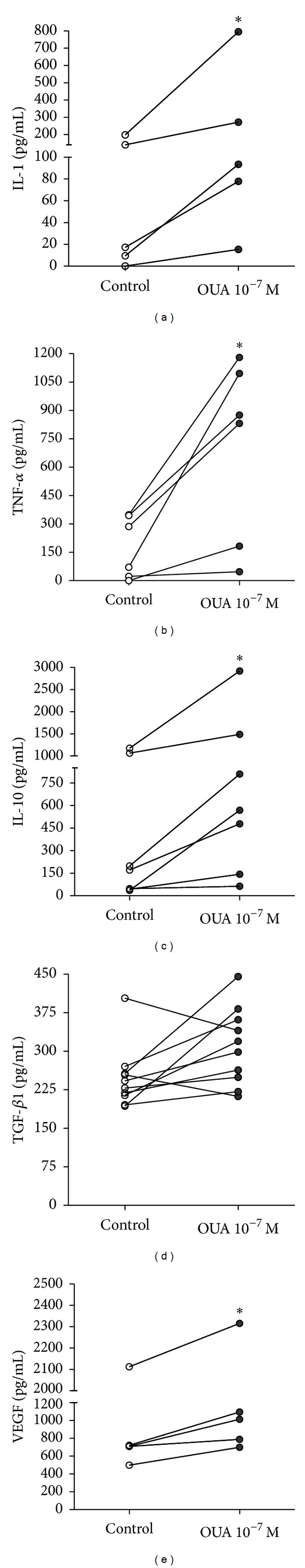
Modulation of cytokine secretion by ouabain. Cells were cultured with 10^−7^ M of ouabain for 24 hours or left untreated. Secretion of IL-1*β*, TNF-*α*, IL-10, TGF-*β*1, and VEGF was measured in these culture supernatants by ELISA. Scatter plots indicate the level of IL-1*β* (a), TNF-*α* (b), IL-10 (c), TGF-*β*1 (d), and VEGF (e) measured in pg/mL and each line represents the data from one specific donor. Each condition in all experiments was performed in duplicate. *P < 0.05 using Wilcoxon matched pairs test.

In accordance with previous studies, ouabain promoted an increase in the secretion of IL-1*β* and TNF-*α* (Figures 6(a) and 6(b)). Nevertheless, the levels of IL-10 and VEGF measured in the supernatants of cells cultured for 24 hours in the presence of ouabain were also higher than control levels (Figures 6(c) and 6(e)). Regarding TGF*β*1, we observed variable results, as ouabain promoted an increase of TGF*β*1 levels in 8 different supernatants and a decrease in 2 others (Figure 6(d)).

### 3.5. Ouabain Increases Dextran-FITC Incorporation

To further investigate the effect of ouabain on monocyte function, the endocytic capacity of monocytes was studied using dextran-FITC particles.

**Figure 7:**
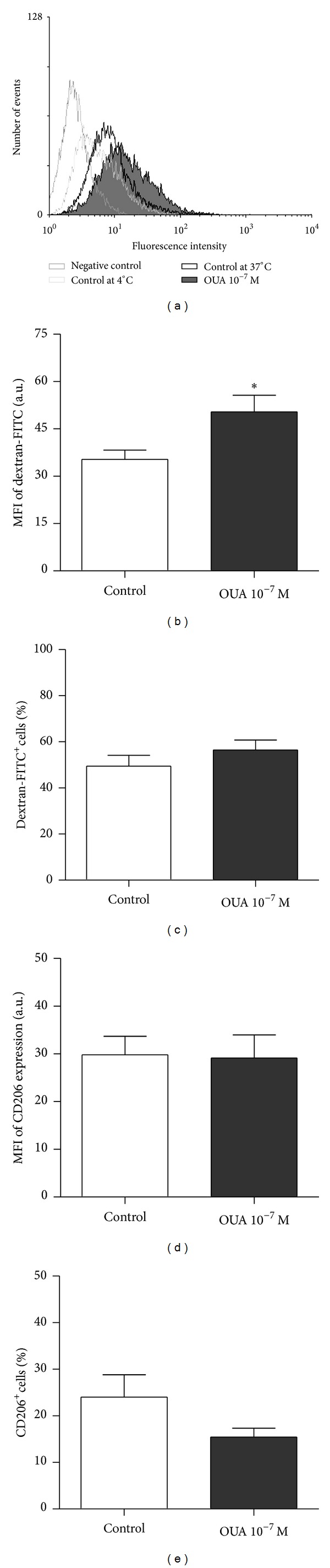
Effect of ouabain on dextran-FITC endocytosis and CD206 expression. Cells were cultured in the absence or presence of 10^−7^ M of ouabain for 24 hours. After 24 hours, these cells were incubated for 1 hour with FITC-conjugated dextran (1 mg/mL) for endocytic assays or incubated for 30 minutes with anti-CD206 PE-Cy5 to evaluate CD206 expression. Fluorescence signals were measured by flow cytometry. (a) Representative experiment showing the patterns of dextran uptake. Other results are expressed as (b) the mean of fluorescence intensity of dextran-FITC ± SEM and (c) the percentage of monocytes engaged in endocytosis—dextran-FITC^+^ cells ± SEM. (d) The mean fluorescence intensity of CD206 ± SEM and (e) the percentage of CD206^+^cells ± SEM. Data are representative of seven endocytic activity assays and four flow cytometric analysis of CD206 expression. *P < 0.05 using two-tailed paired *t*-test.

Our results show an increased dextran-FITC uptake by ouabain-treated monocytes, characterized by an increase in mean fluorescence intensity of these cells (Figure 7(a)). On the other hand, no significant difference was observed on the percentage of dextran-FITC^+^ cells, suggesting that ouabain did not affect the number of monocytes engaged with the endocytosis of these particles (Figure 7(b)).

It has been previously described that the mannose receptor is related to dextran incorporation [25]. However, in our experiments, no increase in CD206 (also known as Macrophage Mannose Receptor) was observed per cell, there was not a significant difference in the number of cells expressing this receptor (Figures 7(c) and 7(d)).

## 4. Discussion 

Monocytes are mononuclear cells produced in the bone marrow and released in the bloodstream, where they represent approximately 10% of the leukocytes present in the peripheral blood [26, 27]. Circulating monocytes are a heterogeneous population and can differ in size, granulosity, and surface molecules expression and may also present distinct migratory and functional properties [28–30]. Monocytes can be further differentiatedinto macrophage and dendritic cells; however, monocytes can be activated and can contribute to immunity without differentiation [31].

A previous study from our group has shown that 10^−7^ M of ouabain decreases the expression of mCD14 on monocytes after 24 hours of culture [21]. This effect could be a consequence of a reduced transcription of CD14 molecule or an increased cleavage of mCD14 into sCD14. If this last hypothesis is true, this could indicate an impact of ouabain on monocyte activation, since sCD14 can be used as a biomarker of monocyte activation [32, 33]. Our present results supported the hypothesis that ouabain promoted a more activated state in monocytes by showing that, after 24 hours of culture, these cells expressed more CD69, HLA-DR, and costimulatory molecules. However, even though an increase of HLA-DR and costimulatory molecules was observed, ouabain did not significantly affect the ability of these monocytes to activate and induce lymphocyte proliferation in allogeneic mixed lymphocyte reactions.

Ouabain was shown to modulate the endocytic ability of monocytes. Our results demonstrated that ouabain increased the amount of dextran particles captured by monocytes, but was not able to affect the number of monocytes that were capable of this endocytosis. The mechanism involved in the increased endocytic ability appears to be independent of CD206, also known as Macrophage Mannose Receptor (MMR), as ouabain did not increase the expression of this molecule. In accordance with this, Kato and coworkers have shown a possible MMR-independent mechanism of dextran-FITC uptake in Langerhans cells, as the uptake of dextran-FITC by these cells was not effectively inhibited by mannose [34]. Thus, it is plausible that ouabain could be increasing the expression of other molecules involved with particles uptake.

Regarding the production of cytokines, an effect of ouabain has been already reported. It has been previously shown that ouabain induced an increase in the production of IL-1*β* and TNF-*α* by mononuclear cells [18–20]. In accordance with these studies, we also observed an increase in IL-1*β* and TNF-*α* using our culture protocol. In addition to that, there was also an enhancement in IL-10 and VEGF secretion; however, we could not observe a clear pattern in TGF*β*1 secretion. We hypothesize that this increase in the production of distinct types of cytokines promotes changes in their balance and may induce diverse responses by different cells. This could explain, at least in part, the complex anti-inflammatory and proinflammatory properties of ouabain.

So far, it is not clear what the exact mechanisms that cause the observed effects are. Similarly to what has already been described in other cell types, ouabain promoted an increase of intracellular calcium levels in monocytes. Moreover, previous results from our group have shown a signaling cascade of ouabain in these cells that involves p38 activation and transactivation of EGFR [21]. Similar signaling pathways have been previously described on myocardial cells. In these cells, ouabain regulated Src-Na^+^,K^+^-ATPase interaction, activated Src, induced transactivation of EGFR, and also activated MAPKs. Activated EGFR is an important element in signal transduction networks, including networks of cytokines [35]. Therefore, it is possible that ouabain could also induce Na^+^,K^+^-ATPase inhibition as well as Na^+^,K^+^-ATPase-mediated signaling in monocytes. Nevertheless, other cell signaling mechanisms induced by ouabain have been shown [35–38] and may also be involved in monocyte ouabain-induced signaling cascade and could lead to the observed effects.

Taken together, these results indicate that ouabain induced a functional state of activation on monocytes, suggesting that it may have an immunomodulatory role on these cells.
